# Combined Effect of Laboratory-Simulated Fire and Chromium Pollution on Microbial Communities in an Agricultural Soil

**DOI:** 10.3390/biology10070587

**Published:** 2021-06-26

**Authors:** Ida Rascio, Maddalena Curci, Concetta Eliana Gattullo, Anna Lavecchia, Mohammad Yaghoubi Khanghahi, Roberto Terzano, Carmine Crecchio

**Affiliations:** 1 Dipartimento di Scienze del Suolo della Pianta e degli Alimenti (Di.S.S.P.A), Università degli studi di Bari “Aldo Moro”, Via G. Amendola 165/A, I-70126 Bari, Italy; ida.rascio@uniba.it (I.R.); maddalena.curci@uniba.it (M.C.); concettaeliana.gattullo@uniba.it (C.E.G.); mohammad.yaghoubikhanghahi@uniba.it (M.Y.K.); carmine.crecchio@uniba.it (C.C.); 2 Dipartimento di Bioscienze, Biotecnologie e Biofarmaceutica, Università degli studi di Bari “Aldo Moro”, Via Orabona, I-70126 Bari, Italy; anna.lavecchia@uniba.it

**Keywords:** Firmicutes, *Paenibacillus*, hexavalent chromium, soil degradation, soil restoration, 16S rRNA sequencing, bioinformatics

## Abstract

**Simple Summary:**

Soil quality and fertility rely on soil microorganisms which contribute to nutrient cycling and plant nutrition. Accidental or intentional fires can almost completely kill soil microbiota and cause soil sterilization. Fires can also destroy soil organic matter (OM), thus causing the release of potentially toxic elements such as Cr that can further disturb soil recolonization by surviving bacteria. The identification of species able to cope with such altered environments is highly relevant to restore soil life in degraded soils and to remediate polluted sites. In this study, we identified soil microorganisms potentially suitable to colonize fire-affected areas and tolerate high concentrations of bioavailable and toxic Cr, and which therefore could be useful for the above-mentioned purposes.

**Abstract:**

Fire events in agricultural soils can modify not only soil properties but also the structure of soil microbial communities, especially in soils containing high concentrations of potentially toxic elements (PTEs). The recolonization of burned soils can in fact favor the proliferation of certain microorganisms, more adaptable to post-fire soil conditions and higher PTE availability, over others. In this study, we simulated with laboratory experiments the microbial recolonization of an agricultural soil containing high Cr concentrations after heating at 500 °C for 30 min, to mimic the burning of crop residues. Changes in soil properties and Cr speciation were assessed, as well as soil microbial structure by means of 16S rRNA gene sequencing. Both altered soil conditions and increased Cr availability, especially Cr(VI), appeared to be responsible for the reduction in species diversity in heated soils and the proliferation of Firmicutes. Indeed, already after 3 days from the heat treatment, Firmicutes increased from 14% to 60% relative abundance. In particular, *Paenibacillus* was the most abundant genus identified after the simulation, with an average relative abundance of 40%. These bacteria are known to be good fire-responders and Cr-tolerant. These results could be useful to identify bacterial strains to be used as bioindicators of altered environments and for the recovery of fire-impacted polluted sites.

## 1. Introduction

Fires affect large areas of land around the world, causing a temporary and localized increase in soil temperatures, often of high magnitude [1]. In the last few decades, uncontrolled fires are becoming increasingly frequent as a consequence of global warming or, in some other cases, of biomass and waste burning on the soil [2]. Additionally, controlled fires are widely used in many common agricultural practices including slash-and-burn farming, land clearing and post-harvest stubble management [3].

High temperatures occurring during (and immediately after) fire events can cause significant changes in soil properties and element biogeochemistry [4]. In particular, the effects of fire on soil properties as well as the short and/or long-term impacts on chemical and microbiological characteristics are strictly related to fire intensity and residence time, which depend on several environmental factors such as fuel distribution, soil mineral composition, climatic conditions, etc. Fire typically increases soil pH and electrical conductivity (EC), reduces the soil organic matter (OM) content, thus altering nutrient cycling (especially P and N cycles), and decreases soil permeability and porosity [4,5].

Fire can also affect the mobility and bioavailability of potentially toxic elements (PTEs), most often as a consequence of changes in soil properties (e.g., OM decomposition and mineral weathering) [6]. In agricultural soils, PTEs’ release and mobilization could represent a potential risk for crop production and consequently for human health [6]. Such a risk is higher for elements such as chromium (Cr), which, depending on the soil redox conditions and the availability of OM, can modify its oxidation state, forming highly mobile and toxic hexavalent species (Cr(VI)) [7].

Soil biological properties have been reported to significantly change after fires as well, since fires can impact (either directly or indirectly) both the composition and the activity of soil microbial communities. Microbial communities can be profoundly altered in the short term through selective heat-induced microbial mortality, whereas medium- and long-term responses are strictly related to indirect effects concerning the changes in the abiotic environment [8,9] and the consequent recolonization by different microbial groups; in fact, several studies have reported that some microbial groups may take advantage of fire-altered conditions, while others may be adversely affected [10].

Firmicutes phylum, for example, was found to be a positive fire-responder, becoming predominant in post-fire soils characterized by low organic carbon content. In the same way, an increase in other fire-responder taxa, such as the spore-forming Actinobacteria and the akinetes producing Cyanobacteria, was observed in different studies [11,12,13]. Some of the strongest fire-responder taxa have also been identified as highly PTE-resistant, such as those belonging to bacterial phyla of Proteobacteria (e.g., genera *Burkholderia*, *Pseudomonas*, *Shewanella* and *Agrobacterium*), Firmicutes (e.g., genera *Serratia*, *Bacillus* and *Exiguobacterium*), and Actinobacteria (e.g., genera *Arthrobacter*) [13].

Most of the studies on the effect of fire on soil’s physico-chemical and microbiological properties have been carried out in forest environments, where these events are more frequent and of higher intensity, while limited information on agricultural soils is available [6]. The soil microbiota plays a key role both in nutrient and PTE cycling in the rhizosphere, with relevant implications for crop productivity and production quality [13]. Fire can induce a positive or negative selective pressure on specific microbial taxa, with consequences not only on plant fitness but also on the soil’s capacity to restore its pre-fire properties [4]. Therefore, it is crucial to assess how fire can shape the composition and functioning of soil microbial communities in agricultural soils, especially in the presence of high concentrations of PTEs.

In this work, 16S rRNA gene sequencing was used for probing the possible changes in bacterial community structure as affected by laboratory-simulated fire events in an agricultural soil containing a high concentration of Cr. We hypothesize that the soil bacterial community diversity and structure change as a consequence of both altered post-fire chemical soil properties and modified speciation and availability of Cr.

## 2. Materials and Methods

### 2.1. Site Description

**Figure 1:**
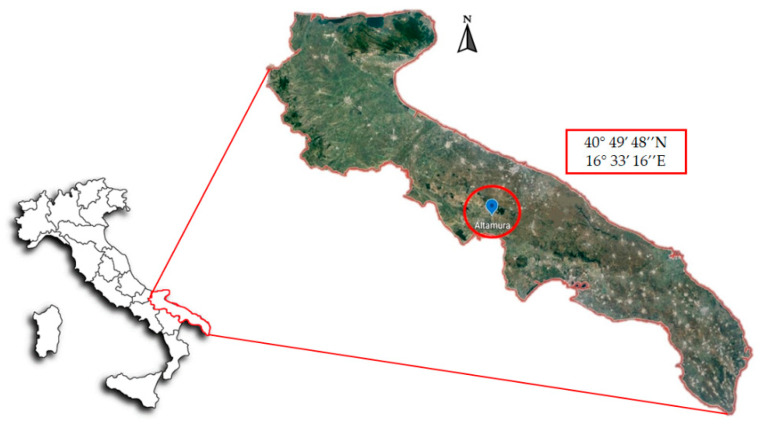
Location of the sampling site with geographic coordinates.

The experiment was conducted in a monoculture of durum wheat (*Triticum durum* Desf.) field in the south of Italy (Apulia Region) near the town of Altamura (Figure 1), amended for more than 10 years by low-quality compost derived from tannery waste sludges, as described by Gattullo et al. (2020) [14]. The soils in this area are classified as Calcaric Leptosols, according to WRB classification [15].

As reported by Gattullo et al. (2020) [14], Cr was the most abundant PTE in the soil, with concentrations up to 5160 g kg^−1^. Anyway, the high OM content (about 220 g kg^−1^) formed strong complexes with Cr and other PTEs, thus immobilizing them and making them not available for plant uptake, and not affecting wheat production [16]. Additionally, the high OM content of soil hindered Cr oxidation; therefore, Cr was present only in the reduced and less mobile form (Cr(III)) [14].

### 2.2. Soil Sampling and Characterization

Three soil sub-samples were collected at 0–10 cm depth in an area of approximately 4 m^2^ and were carefully homogenized. A portion of soil was immediately stored at 4 °C for the subsequent microbial extraction, while the remaining part was air-dried, sieved at 2 mm, and then characterized for pH, EC, organic C content, total N content, available P, total CaCO_3_ and exchangeable bases, following the standard methodologies of soil analysis [17]. The total PTE content was determined by ED-XRF (NITON XL3t GOLDD with laboratory stand, Thermo Scientific) following the procedure reported in Gattullo et al. (2020) [14]. Chromium(VI) was determined after the alkaline digestion of soil samples [18], while the exchangeable Cr(VI) was extracted by shaking the soils for 30 min with a 5 mM K_2_HPO_4_/KH_2_PO_4_ buffer solution (pH 7.2) at a ratio of 1:4 (soil: buffer solution, *w/v*), as reported in Bartlett and James, 1996 [19]. The Cr(VI) estimation in both extracts (total Cr(VI) and exchangeable Cr(VI)) was performed by the colorimetric assay with diphenylcarbazide [20]. The detection limit of the assay is 0.0052 mg L^−1^ [19], corresponding to about 0.2 μg Cr(VI) g^−1^ of dry soil for the total Cr(VI), and 0.02 μg Cr(VI) g^−1^ of dry soil for the exchangeable Cr(VI).

PTE plant-available fraction (DTPA-extracts) was also determined by extracting the soil sample with a diethylenetriaminepentaacetic acid (DTPA) solution (0.005 M DTPA, 0.01 M CaCl_2_, 0.1 M triethanolamine, pH = 7.3) [21] and analyzing the extracts by ICP-OES (Thermo iCAP 6000 series, Thermo Fisher Scientific Inc., Waltham, MA, USA).

### 2.3. Soil Thermal Treatment

Fire was simulated in the laboratory by heating the soil in a muffle furnace (Nabertherm, B180). Muffle furnace heating is one of the most common strategies to simulate fire events in the lab, as reported by Pereira et al. (2019) [22]. Two hundred grams of soil was placed in a 20 cm diameter ceramic crucible, creating a soil layer of about 0.7 cm thickness to allow a homogeneous heat transfer through the sample. Soil was heated up to 500 °C for 30 min. Such a temperature was selected after performing preliminary thermogravimetric analyses (not reported) showing that at 500 °C almost all the soil OM was lost [4]. Heating time was selected according to studies on agricultural soils where such high temperatures were recorded for about 30 min after burning crop residues on soil [23]. Three replicates were set up and, for each of them, soil characterization was carried out as described in Section 2.2.

### 2.4. Extraction of Bacterial Communities and Soil Inoculation

About 100 g of unheated soil was weighed and homogenized in 900 mL 25% sterile Ringer solution and 100 mL sodium pyrophosphate (Na_4_P_2_O_7_ 1,8%). Ringer solution was prepared by solubilizing in deionized water NaCl (0.225% *w/v*), KCl (0.0105 % *w/v*), CaCl_2_ (0.0045% *w/v*), NaHCO_3_ (0.005% *w/v*) and citric acid (0.0034% *w/v*) at pH = 7.0 ± 0.2 [24]. The solution was autoclaved for 15 min at 121 °C before use. Microbial community desorption from soil particles was performed by sonication for 2 min and subsequent storage at 4 °C for 15 min to let soil particles sediment. Serial dilutions (10^−4^, 10^−5^ and 10^−6^) of the aqueous phase were performed and poured on nutrient agar plates after addition of cycloheximide (1% *w/v*) to determine the number of CFU. The plates were then incubated at 30 °C for 48 h. Since after 48 h the number of grown colonies for each plated dilution was very low, about 80 mL of 10^−1^ dilution was used for the inoculation of 100 g aliquots of the 500 °C heated soil sample. Each aliquot was then incubated in sterile glass flasks closed with a screw cap, and kept for 3, 7 and 14 days (T3, T7 and T14) in a growth chamber at 23 °C. For each incubation time, three experimental replicates were set up. Inoculation of bacterial community extracted from untreated soil was performed to simulate a recolonization of the soil after burning, since heating treatment at 500 °C causes soil sterilization.

### 2.5. Soil DNA Extraction, 16S rRNA Sequencing and Bioinformatics Analysis

Soil DNA was extracted from 0.5 g of both unheated soil (Control) and 500 °C-heated and inoculated soil aliquots (T3, T7 and T14) by using the soil DNA extraction kit (MP Biomedicals™ FastDNA™ SPIN Kit) and following the manufacturer’s instructions. The quality and concentration of the extracted DNA were verified with the NanoDrop spectrophotometer (ND-1000, EuroClone, Italy). The DNA was then concentrated up to 20 ng µL^−1^ by SpeedVac concentrator (Savant DNA120, ThermoScientific) and stored at −20 °C prior to the sequencing procedure. Universal primers: 341F (5′–CCTACGGGNGGCWGCAG–3′) and 805R (5′–GACTACHVGGGTATCTAATCC–3′), based on the V3 and V4 hypervariable region of the 16S rRNA, were used for the detection of *Bacteria*, and the sequencing procedure was performed by using an Illumina MiSeq next-generation sequencer (Illumina, San Diego, CA, USA). The sequencing was carried out at the IGA Technology Service (Udine, Italy) (https://igatechnology.com, accessed 7 January 2020).

Raw reads produced by Illumina sequencing were processed using the Metagenomic Rast Server (MG-RAST) (http://metagenomics.anl.gov, accessed on 23 April 2020) [25]. Raw data were uploaded as FASTQ files and subjected to quality control, which includes the removal of artificial duplicate reads, quality-based read trimming, and length-based read trimming. The sequences were then clustered into operational taxonomical units (OTUs) at 97% similarity and then subjected to taxonomic assignment using the Ribosomal Database Project (RDP) Naïve Bayesian classifier.

Sequence data were deposited at the National Center for Biotechnology Information (NCBI) and available under the SRA accession: PRJNA723052.

Relative abundances of each taxon were calculated as a percentage of the total number of sequences for each sample; only the relative abundances greater than 1% are shown in the text.

### 2.6. Statistical Analysis

The *α*-diversity indices were statistically analyzed by one-way analysis of variance (ANOVA), and means were compared by the Student–Newman–Keul (SNK) test at *p* ≤ 0.05 using the SPSS package (SPSS Inc., v.24, Chicago, IL, USA).

The Bray–Curtis dissimilarity matrix was used for the analysis of similarity (ANOSIM) to highlight the differences among bacterial composition in the investigated soils.

The *β*-diversity was evaluated by Principal Coordinate Analysis (PCoA) to compare and plot the structure of all samples. Moreover, Canonical Correspondence Analysis (CCA) was used to relate the relative abundances of the phyla (>1%) to some selected environmental variables in a two-dimension graphic. Finally, SIMilarity PERcentage (SIMPER) analysis was carried out to identify the OTUs responsible for the differences between T3, T7 and T14 samples. Multivariate statistical approach (ANOSIM, PCoA, CCA and SIMPER) and Ward’s clustering method were performed using PAST 3.17 software.

## 3. Results

### 3.1. Soil Chemical Characterization

Soil chemical analyses, performed both on unburned soil and 500 °C thermally treated soil, revealed that the heating treatment significantly altered the soil chemical properties (Table 1).

After the thermal treatment, pH and even more EC increased compared to untreated soil. The organic C content was strongly reduced and, consequently, the value of OM content decreased from 234 g kg^−1^ in unburned soil to 24 g kg^−1^ in burned soil. The heating treatment also reduced the total N content by about 80%, while it increased the available P by more than twice and total carbonates by 18%. The concentrations of exchangeable base cations in the unburned soil followed the sequence Ca^2+^ > K^+^ > Mg^2+^ > Na^+^, while after the 500 °C thermal treatment they followed the sequence Ca^2+^ > Mg^2+^ > K^+^ > Na^+^. In particular, K^+^ and Ca^2+^ concentrations decreased in the heated soil.

The total concentrations of Cu, Pb, Zn and Cr increased after the thermal treatment by 50%, 55%, 44% and 11%, respectively. Total PTE concentration increased as a consequence of OM loss. Available Pb and Cu remained almost similar after the thermal treatment, while potentially available Zn decreased and available Cr dramatically increased. In addition, the concentration of Cr(VI), which is the most mobile and toxic form of Cr, changed from undetectable values in the untreated soil to 152 mg kg^−1^ in the heated soil. Moreover, about 22% of Cr(VI) was exchangeable, thus potentially available for plants and microorganisms. The soil chemical properties of burned soil did not change during the different incubation times (3, 7 and 14 days), as expected (data not shown).

### 3.2. Soil Bacterial Communities and Correlation with Physico-Chemical Soil Properties

**Figure 2:**
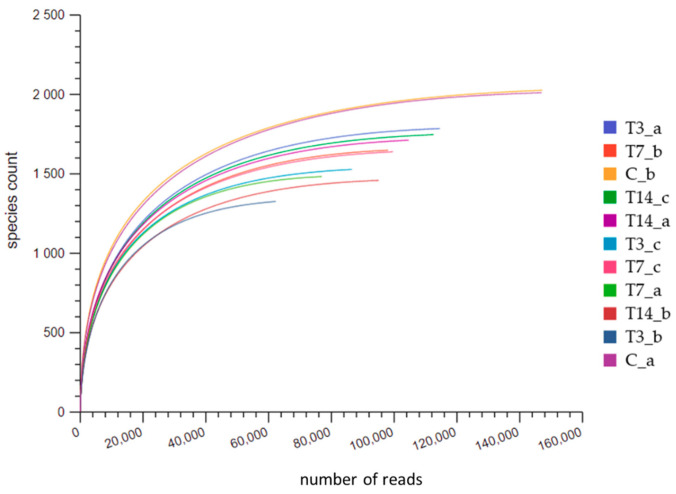
Rarefaction curves relative to the number of sequences and OTUs associated with each soil sample. C are the control unheated samples; T3, T7, T14 are the thermally treated samples after 3, 7 and 14 days of incubation, respectively; a, b, c are the replicates.

Illumina sequencing produced a total of 1,144,661 raw reads, reduced to 953,904 reads after quality control. The rarefaction curves (Figure 2), drawn by plotting the number of sequences and the OTUs associated with each sample, showed that the coverage of sequencing was reasonable. Nevertheless, considering the different size of sequences among samples, a reduced dataset was built by randomly selecting 52,517 sequences for each sample, corresponding to the lowest number of sequences obtained in the sample T3_c.

The C_a and C_b samples (two replicates of the control unheated sample) had the highest number of sequences, compared to T3, T7 and T14 samples.

Consequently, the α-diversity indices (Shannon, Simpson 1-D, Evenness) (Table 2) were calculated for all the samples under investigation on the normalized dataset. The highest number of OTUs, as well as the highest values of Shannon and Evenness indices, were observed in the control sample, thus pointing out a significant difference compared to T3, T7 and T14 samples.

The taxonomic composition showed that the identified sequences were related to 25 phyla, 49 classes, 109 orders, 253 families, 793 genera and 3058 species within all the samples under investigation (C, T3, T7 and T14).

**Figure 3:**
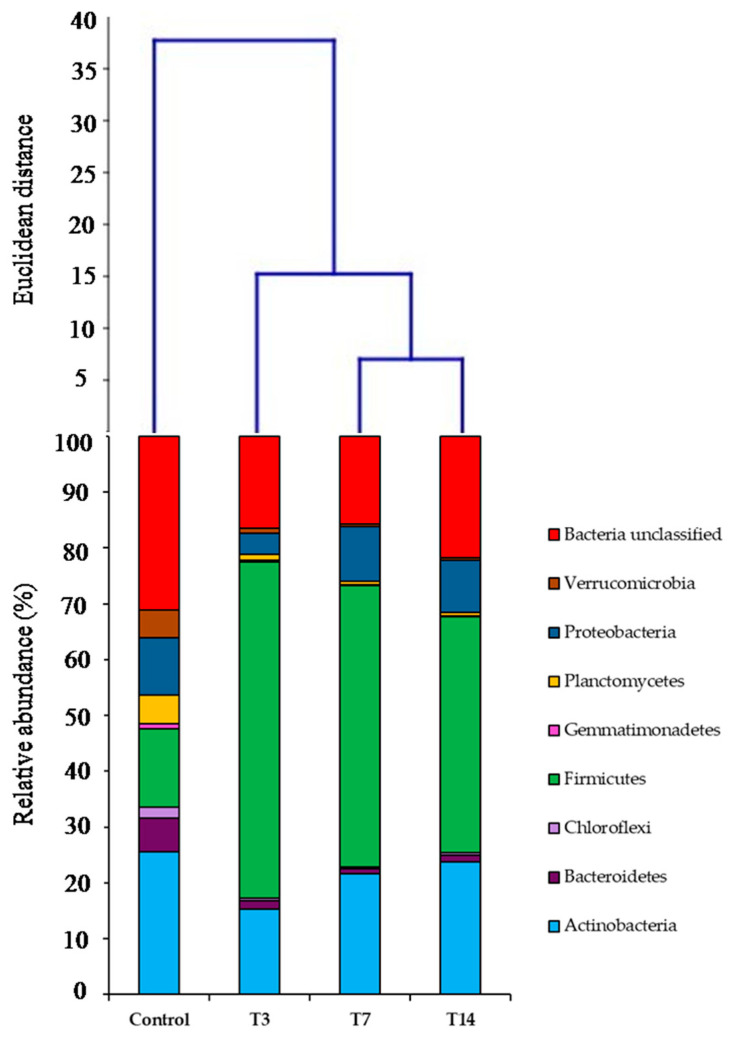
Relative abundances of the dominant bacterial phyla (>1%) and cluster analysis of control, T3, T7 and T14 samples using Ward’s method.

Eight phyla were considered as the most abundant, represented by more than 1% of bacterial total sequences: Actinobacteria, Bacteroidetes, Chloroflexi, Firmicutes, Gemmatimonadetes, Planctomycetes, Proteobacteria, Verrucomicrobia, and unclassified bacteria (Figure 3).

The analysis of the taxonomic profile revealed a significant difference among the soils under investigation (Figure 3), which was considerably evident already at the phylum level. The difference among all the samples was confirmed by the ANOSIM global test of the relative abundances, by comparing group similarities (R = 0.61 and *p* = 0.0012). Additionally, the pairwise comparison highlighted that the control sample (C) was very different compared to T3, T7 and T14 samples (R = 1 for C vs. T3, T7, T14, each); on the other hand, no significant differences among T3, T7 and T14 samples (R = −0.03 for T3 vs. T7; R = 0.70 for T3 vs. T14; R = 0.14 for T7 vs. T14) have emerged.

Firmicutes relative abundance increased up to 60% after 3 days of incubation (T3), whereas it represented only 14% of the total sequences in the control. Moreover, Firmicutes relative abundance slightly decreased 7 and 14 days after inoculation down to percentages of 50% and 42%, respectively. On the other hand, Actinobacteria and Proteobacteria relative abundances decreased after 3 days of incubation, compared to the control, while after 7 and 14 days from the inoculation they increased, reaching approximately the initial values (about 25% for Actinobacteria and 10% for Proteobacteria). A low percentage of the sequences of the control was associated with Bacterioidetes, Planctomycetes, Verrucomicrobia, Chloroflexi, and Gemmatimonadetes; these phyla rapidly and strongly decreased after 3 days of incubation (T3) and continued to decrease after 14 days (T14), when they showed relative abundances of 1%, 0.5%, 0.4% (both Verrucomicrobia and Chloroflexi) and 0.1%, respectively. Euclidean distances among the control, T3, T7 and T14 soil samples were computed using Ward’s clustering algorithm. In this regard, treatments clustered into different groups in terms of bacterial communities at the phylum level (relative abundance > 1%), in which T3 treatment has less distance to the control compared to T7 and T14 treatments (Figure 3).

**Figure 4:**
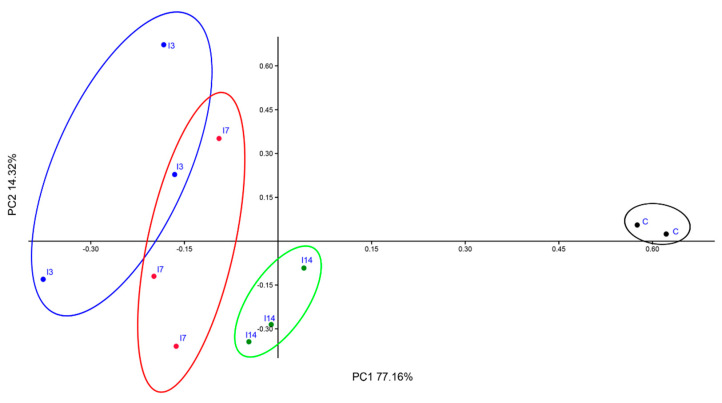
PCoA plot of control (C), T3, T7 and T14 soil bacterial communities.

PCoA better illustrates the differences between the structure of bacterial communities associated with the control (C), T3, T7 and T14 soil samples (Figure 4). The two-dimensional plot revealed a clear segregation of C from T3, T7 and T14 samples. The first axis, accounting for about 77% of the variance, highlighted the differences between the T3, T7 and T14 and the control soil. The second axis (~14%) separated C from T14 well.

**Figure 5:**
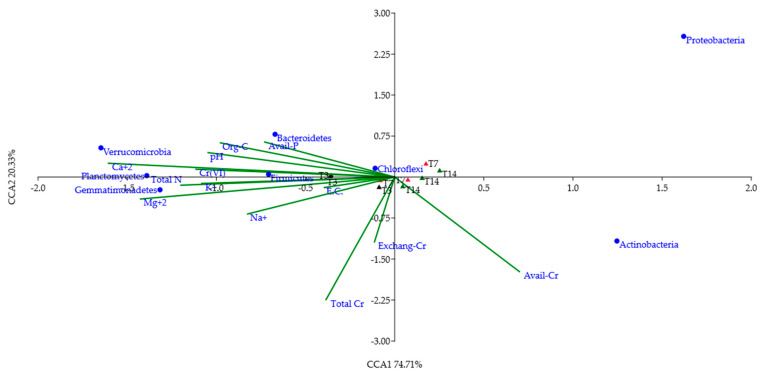
CCA biplot showing the relationships between the most abundant phyla (>1%) and selected chemical properties of T3, T7 and T14 samples.

Canonical correspondence analysis (CCA) aimed at explaining the relationships between the most abundant phyla (relative abundance ≥ 1%) and selected environmental variables (soil parameters) of the T3, T7 and T14 samples. The selected soil parameters were: pH, EC, base cations, organic C, total N, available P, total Cr, DTPA-extractable Cr, total Cr(VI), and exchangeable Cr(VI). Most of the total variance (95%) was accounted by seven axes and explained by the first two components (Figure 5). The first axis, accounting for about 75% of variance, clearly discriminated T3 from T7 and T14. A further division within these three main clusters accounted for about 20% of variance by the second axis.

With the exception of Actinobacteria and Proteobacteria, all other phyla were strictly correlated to most of the considered environmental parameters, Cr(VI) included. According to CCA (Figure 5), *Firmicutes* was highly related to Cr(VI) content and other soil chemical properties such as pH, organic C, total N, available P, Ca^2+^. On the other hand, Bacteroidetes, Verrucomicrobia, Gemmatimonadetes, Chloroflexi and Planctomycetes were less influenced by these soil properties, while Actinobacteria and Proteobacteria were not influenced by any variable. Looking at the soil microbiota at the three different times of incubation, T3 samples appeared related to Cr(VI) concentration, soil pH, organic C, total N, available P, Ca^2+^, Mg^2+^ and K^+^, whereas T7 and T14 samples were mainly related to total Cr, available Cr and exchangeable Cr(VI).

The SIMPER analysis (Table 3) on the relative abundance of OTUs (genus level) revealed that OTUs belonging to Firmicutes, Actinobacteria and Proteobacteria phyla were those mainly responsible for differences between T3, T7 and T14. Particularly, OTU belonging to *Paenibacillus* genus contributed to ~40% of differences.

## 4. Discussion

Chemical analyses performed on both unheated and heated soil revealed that heating treatment significantly altered most of the soil properties (Table 1). The increase in pH observed in burned soil was ascribable to the loss of organic acids, the release of oxides, hydroxides, carbonates and cations through ash particles, and the displacement of H^+^ from the exchange sites of clay minerals [26,27]. The soil EC considerably increased after heating because of the release of soluble inorganic ions deriving mainly from the combustion of OM, but also from the exchange complexes [28]. The increase in soil salinity can enhance the PTEs’ mobility due to the competition between salt-derived ions and PTE ions for the adsorption sites, as well as to PTE complexation by salt-derived anions [29]. The organic C content, as well as the total N concentration, decreased after thermal treatment by about 90% and 80%, respectively. This was due to the mineralization of the OM, which is almost total at 460 °C [30]. Furthermore, the OM combustion released inorganic P, as proved by the doubling of the available P concentration in heated soil compared to unheated soil. All these modifications reveal the positive effects of fire in regard to the nutrient release, at least in the short term (14 days).

The PTE total concentrations after the thermal treatment increased by a minimum of 11% (for Cr) to a maximum of 55% (for Pb). However, the hazardousness of PTEs is correlated more to their mobility and bioavailability, rather than to their total concentrations. Therefore, the quantification of the PTE plant-available fraction (estimated through DTPA-extraction) is a better index of potential risk for microorganisms and crops and, via the food chain, for human health. Zinc DTPA-extractable fraction decreased after the thermal treatment, Cu and Pb remained almost unchanged, while the DTPA-extractable fraction of Cr increased from 0.3 to 105 mg kg^−1^. Indeed, in the unheated soil, Cr was almost completely immobilized by OM, as reported by Gattullo et al. (2020) [14]. After soil burning, part of the Cr complexed or entrapped by OM was released, thus explaining the sharp increase in its available fraction. Another dangerous transformation caused by fire in soil was the oxidation of Cr(III) to Cr(VI), in agreement with other pieces of evidence reported in the literature [7,31]. As suggested by Panichev et al. (2008) [7], Cr oxidation might have been favored by the presence of carbonates released by fuel combustion. In the soil under investigation, the concentration of total carbonates was very high already before the fire, then it further increased after the laboratory fire simulation (Table 1). Additionally, Fe and Mn oxyhydroxides’ transformation might have also contributed to the Cr oxidation process [31]. The Cr (VI) concentration after the thermal treatment was 152 mg kg^−1^, a level considerably higher than the safety threshold established for agricultural sites in Italy (2 mg kg^−1^; Italian Legislative Decree n. 152/2006) [32]. About 20% of the total concentration of Cr(VI) was exchangeable and therefore potentially available for plant and microbe uptake.

The soil chemical properties did not change during the incubation period after bacteria inoculation (data not shown), in agreement with evidence reported in the literature. Indeed, the restoration of physico-chemical soil characteristics after a fire requires from a few months to several years, at least in an open system [4,11,33]. In the present study, the soil was incubated for 14 days in a closed system, thus the occurrence of chemical modifications was scarcely probable.

Heat can directly affect the survival and the recolonization of soil microbial communities since the temperatures occurring during a fire event often considerably exceed those for killing most of the living organisms [4]. Previous field-scale experiments have revealed that high temperatures (≥500 °C), reached during high-severity fires, are generally restricted to a soil depth of no more than 1 cm, with a residence time from some minutes up to several hours, depending on fuel density and distribution [4,13,34]. Fires are often extremely heterogeneous, depending on localized fuel loads and wind strength and direction, which result in large patches of unburned and moderately burned areas. Consequently, the microbial recolonization processes are generally driven by the bacterial communities of the unburned areas or of the deeper horizons/layers, in the case of post-fire plowing [35].

Indirect effects on microbial phylogenetic and taxonomic composition are related to different soil properties such as soil moisture, quantity and quality of organic carbon and nutrient cycling [9,36,37]. Moreover, fire severity could lead to different recolonization pathways, thus favoring the growth of some bacterial taxa rather than others. Among the factors influencing the bacterial recolonization processes, PTEs’ concentration and speciation can also play a crucial role in selecting specific taxa able to survive and promote environmental restoration.

Alpha diversity indices (i.e., species richness and evenness within a sample) are generally related to the ecosystem stability and functionality [38]; in particular, the Simpson 1-D index is weighted towards the abundances of the most common species, whereas the Shannon index is more influenced by species richness [39]. In the present study, the total species richness was significantly altered by post-fire changes in the abiotic environment. The higher diversity was found in the control (microbiota extracted from the unheated soil) compared to T3, T7 and T14 samples (microbiota extracted from heated soil inoculated and incubated for 3, 7 and 14 days) (Table 2). The reduction in diversity in T3, T7 and T14 samples was probably due to some taxa’s inability to physiologically cope with abiotic stressors. In T3, T7 and T14 samples, in fact, the combination of high Cr(VI) content (152 mg kg^−1^, with about 20% in the exchangeable form) and fire-altered soil chemical properties probably induced a negative selective pressure on a number of taxa, thus reducing the microbial diversity. This evidence matched with the findings reported in other studies, where both the contribution of wildfires and soil pollution on bacterial group selection are widely discussed [12,13,38,40].

Cluster analysis showed more shifts in the structure of bacterial communities in T7 and T14 treatments than in T3 samples, indicating that they were more sensitive to these variations. In this regard, taxonomic composition revealed a significant shift from Actinobacteria in the control to Firmicutes in T3, T7 and T14 samples (Figure 3). However, Actinobacteria relative abundances increased again after 14 days of incubation. A similar trend was observed for Proteobacteria. The shift at phylum-level suggests that Firmicutes can immediately (after 3 days incubation) deal with post-fire changes, including Cr oxidation and increasing bioavailability, as well as alterations in soil chemical properties (i.e., pH, EC, organic C, total N, available P, etc.). Indeed, Firmicutes, Actinobacteria and Proteobacteria were detected in different investigations where both PTE pollution and wildfire severity were considered as drivers of soil microbial diversity [12]. Miranda et al. (2018) [40] focused on responses of soil microbial communities after the application of tannery sludge for seven years. They found that Cr content in association with increased pH and organic C were the key variables that influenced the soil microbial structure. In particular, the amendment of soil with 10 and 20 Mg ha^−1^ of composted tannery sludge promoted a shift in bacterial community composition compared to the control unamended soil. The analysis of sequences revealed that Actinobacteria, Proteobacteria and Firmicutes were the most abundant phyla in amended soils, suggesting that these bacterial groups have a great tolerance to altered soil conditions and Cr contamination [40]. Noteworthy, it should be remembered that Firmicutes and Actinobacteria include endospore-forming bacteria, which are able to survive under environmental stresses. These groups were also found in different studies focusing on high-severity fire occurrence [13], thus confirming their ability to survive and colonize extremely altered environments.

The Planctomycetes, Chloroflexi and Verrucomicrobia relative abundances decreased after 3, 7 and 14 days of incubation compared to the control. In accordance with Miranda et al. (2018) [40], Planctomycetes relative abundance probably decreased as a consequence of the increase in soil pH and total Cr concentration, whereas the decrease in Chloroflexi and Verrucomicrobia was probably due to the selected temperature (500 °C) and residence time (30 min), as these parameters are generally ascribed to high-severity fires [12].

All the above-discussed results were supported by the PCoA plot showing a clear separation between the bacterial communities of the control and those of T3, T7 and T14 samples (Figure 4). Furthermore, CCA (Figure 5) showed the associations between the most abundant phyla (with relative abundances > 1%) detected in T3, T7 and T14 samples and the chemical variables which were considered relevant for taxa selection.

At first glance, a correlation between T3 samples, Cr(VI) content and some of the main soil chemical parameters (pH, EC, organic C, available P, total N and base cations) was observed. This could be explained by considering that 3 days after the microbial inoculation, bacterial groups, able to grow in extreme environmental conditions, started to colonize the heated soil. Generally, as reported by Saenz de Miera et al. (2020) [12], taxa with oligotrophic strategies as well as spore-forming species are good fire-responders. Among these taxa, Firmicutes are spore-forming bacteria and have been noted to grow after high-severity fire disturbance [13]. These findings confirm the idea that nutrient deficiencies and low organic C content in soil lead to an increase in the relative abundance of oligotrophs. Previous studies have revealed that, in the case of environmental stress due to and/or resulting in low-resource concentrations in soils, the oligotrophs prevail over the copiotrophs [41,42]. Wang et al. (2019) [43] and Yaghoubi et al. (2020) [44] also reported a negative relationship between the relative abundance of oligotrophic taxa and C substrates in soil. The Firmicutes phylum was also identified as PTE-resistant; in particular, total Cr concentration has been shown to affect the relative abundances of different genera belonging to Firmicutes [40]. A positive correlation among some altered soil chemical properties, Cr(VI) and Firmicutes was also evident in CCA (Figure 5), especially in T3 soil, and is in line with the above-mentioned studies.

The CCA (Figure 5) evidenced that after 7 and 14 days of incubation, soil samples are more influenced by the total Cr content, the exchangeable Cr(VI) (Exchang-Cr) and the DTPA-extractable Cr (Avail-Cr). The differences observed in CCA among the T3, T7 and T14 soil samples suggest that, after 14 days of incubation, the highly PTE-tolerant microbial groups colonizing the soil samples may have promoted the soil recovery to pre-fire conditions.

Bacteroidetes, Verrucomicrobia, Gemmatimonadetes and Chloroflexi were less influenced by the selected chemical variables, while Proteobacteria and Actinobacteria were not influenced by such environmental parameters. In this regard, no matches were found in literature studies. Finally, the SIMPER analysis (Table 3) identified the genus *Paenibacillus*, belonging to the *phylum Firmicutes*, as the main factor responsible for the dissimilarity between T3, T7 and T14. In particular, *Paenibacillus* contributed to the community dissimilarity with a percentage of about 40%. This result is in line with the work of Miranda et al. (2018) [40], as *Paenibacillus* was found to be highly resistant to high Cr concentrations and is considered a good bioindicator of Cr pollution.

## 5. Conclusions

In this work, a laboratory-scale approach was used to study the effects of high temperatures on the chemical and microbiological properties of a Cr-polluted agricultural soil.

Despite the high Cr total concentration, previous studies [12,13,45,46] have shown limited environmental risks in the investigated soils because of the huge OM content that does not allow Cr mobilization and the oxidation of Cr(III) to more toxic Cr(VI). Fire occurrence (accidental or intentional) might pose a serious risk for the environment and human health since high temperatures could cause OM depletion, leading to Cr(III) oxidation and mobilization. Such remobilization could negatively affect not only crops but also soil microorganisms and therefore soil quality.

Heating treatment considerably altered soil physico-chemical properties (pH, EC, total N, organic C, available P, base cations) and changed Cr speciation into more mobile and potentially available chemical forms, including the formation of significant amounts of Cr(VI), as well as a different colonization capacity of microbial communities. Soil microbial analyses revealed, in fact, a predominance of Firmicutes (*Paenibacillus* genus) in response to altered soil conditions. Specifically, 3, 7 and 14 days after the inoculation of autochthonous bacterial communities, Firmicutes relative abundances were about 60%, 50% and 42%, respectively. This phylum is known to include good fire-responder species as well as PTE-resistant microorganisms. In particular, Cr concentration appeared to affect the relative abundance of the genus *Paenibacillus*, which is considered a good bioindicator of Cr contamination. Indeed, SIMPER analysis performed on T3, T7 and T14 samples revealed that *Paenibacillus* made the greatest contribution to the community dissimilarity with a percentage of about 40%.

The results reported here could constitute a starting point for the identification of biological indicators of Cr pollution in soils after fire events and their isolation both for bioremediation purposes and post-fire recovery of fire-impacted polluted sites.

## Figures and Tables

**Table 1:** Chemical properties and potentially toxic element (PTE) concentrations in the unheated and heated soils (mean value ± standard deviation, *n* = 3). Only one determination was performed for the basic chemical properties of the unheated soil, being a composite sample.

Chemical Characteristics		Unheated Soil	Heated Soil
pH (H_2_O)		7.5	8.1 ± 0.1
EC (mS cm^−1^)		0.2	1.8 ± 0.1
Total N (g kg^−1^)		15	2.6 ± 0.2
Available P (mg kg^−1^)		181	397 ± 4.4
Organic C (g kg^−1^)		136	14 ± 1
Total CaCO_3_ (g kg^−1^)		202	240 ± 30
Ca^+2^ (cmol_(+)_ kg^−1^)		47	39 ± 2
Mg^+2^ (cmol_(+)_ kg^−1^)		1.9	1.8 ± 0.5
Na^+^ (cmol_(+)_ kg^−1^)		0.1	0.3 ± 0.1
K^+^ (cmol_(+)_ kg^−1^)		2.2	0.8 ± 0.1
Cu	Total (mg kg^−1^)	134 ± 5	201 ± 11
	Available (mg kg^−1^)	14.0 ± 0.7	8.8 ± 0.3
Pb	Total (mg kg^−1^)	114 ± 3	177 ± 5
	Available (mg kg^−1^)	5.0 ± 0.3	8.3 ± 0.5
Zn	Total (mg kg^−1^)	1270 ± 10	1834 ± 23
	Available (mg kg^−1^)	208 ± 25	59 ± 6
Cr	Total (mg kg^−1^)	5160 ± 35	5715 ± 13
	Available (mg kg^−1^)	0.30 ± 0.03	105 ± 9
Cr(VI) (µg g^−1^)		b.d.l.	152 ± 44
Exchangeable Cr(VI) (µg g^−1^)		b.d.l.	34 ± 4

b.d.l.: below detection limit.

**Table 2:** Species richness and diversity indices of bacterial communities of control (C), T3, T7 and T14 soil samples.

Samples	Reads	Good Quality Sequences	Observed Species *	Shannon *	Simpson 1-D *	Evenness *
C	147,107 ± 100	112,810 ± 54,505	1669 ± 7.78 ^a^	2.72 ± 0.05 ^a^	0.82 ± 0.01 ^a^	0.37 ± 0.02 ^a^
T3	87,810 ± 26,178	68,503 ± 15,883	1286 ± 192.22 ^b^	1.87 ± 0.21 ^b^	0.68 ± 0.07 ^a^	0.16 ± 0.03 ^b^
T7	91,530 ± 10,324	81,755 ± 11,929	1317 ± 73.43 ^b^	2.28 ± 0.10 ^b^	0.80 ± 0.03 ^a^	0.23 ± 0.02 ^b^
T14	104,142 ± 393	92,503 ± 6150	1384 ± 129.06 ^b^	2.20 ± 0.30 ^b^	0.79 ± 0.06 ^a^	0.22 ± 0.07 ^b^

* Values, calculated on a normalized dataset (52,517 sequences), are means ± standard deviation of 3 replicates for each sample, except for C (*n* = 2); data with different letters in each column are significantly different, according to SNK test at *p* < 0.05.

**Table 3:** Similarity percentage analysis (SIMPER) showing (i) the relative abundance of each identified genus and the corresponding phylum, (ii) the contribution to the total diversity and, (iii) the cumulative contribution to the average similarity. Contributions below 3% are not shown.

Phylum	Genus	Abundance (%)			Contribution %	Cumulative %
		T3	T7	T14		
Firmicutes	*Paenibacillus*	46.50	35.65	29.93	39.06	39.06
Firmicutes	*Cohnella*	3.71	6.01	5.07	8.55	47.61
Actinobacteria	*Arthrobacter*	5.22	3.39	1.49	7.88	55.49
Actinobacteria	*Nocardioides*	1.49	3.96	3.75	7.52	63.01
Proteobacteria	*Rhizobium*	0.40	2.64	1.73	5.26	68.28
Actinobacteria	*Geodermatophilus*	0.67	1.87	2.53	5.15	73.42
Actinobacteria	*Pimelobacter*	0.37	2.65	1.87	4.47	77.89
Actinobacteria	*Kocuria*	0.29	2.82	0.07	4.43	82.31
Firmicutes	*Bacillus*	2.38	3.04	1.60	3.98	86.30
Proteobacteria	*Ensifer*	0.04	1.52	0.97	3.28	89.58

## Data Availability

Sequence data were deposited at the National Center for Biotechnology Information (NCBI) and available under the SRA accession: PRJNA723052.
